# Smoking and Depth-Related Anaerobic Bacteria in Endodontic–Periodontal Lesions: A Pilot Study

**DOI:** 10.3390/ijerph23070860

**Published:** 2026-06-30

**Authors:** Cássio Vicente Pereira, Natália Galvão Garcia, Douglas Campideli Fonseca, Pedro Gustavo Machado, Michele de Fátima Rezende, Sarah Ferreira Mattos Alcântara, Eric Francelino Andrade, Luciano José Pereira

**Affiliations:** 1 School of Dentistry, Centro Universitário de Lavras (UNILAVRAS), Lavras 37203-593, Minas Gerais, Brazil; cassio@unilavras.edu.br (C.V.P.); nataliagalvao@unilavras.edu.br (N.G.G.); douglas@unilavras.edu.br (D.C.F.); 2 Department of Medicine, Faculty of Health Sciences (FCS), Federal University of Lavras (UFLA), Lavras 37203-202, Minas Gerais, Brazil; pedro.machado2@estudante.ufla.br (P.G.M.); michele.rezende1@estudante.ufla.br (M.d.F.R.); sarah.alcantara@estudante.ufla.br (S.F.M.A.); eric.andrade@ufla.br (E.F.A.)

**Keywords:** endodontic–periodontal lesions, smoking, subgingival microbiota, anaerobic bacteria, periodontal pocket depth, *Porphyromonas gingivalis*, *Tannerella forsythia*

## Abstract

**Highlights:**

**Public health relevance—How does this work relate to a public health issue?**

- Endodontic–periodontal lesions are clinically relevant oral infections that may contribute to periodontal destruction, tooth loss, and increased treatment complexity in adult patients.
- This study addresses smoking as a public health-related behavioral factor that may alter microbial patterns in periodontal pockets affected by combined endodontic–periodontal disease.

**Public health significance—Why is this work of significance to public health?**

- The findings suggest that smokers and non-smokers may show different depth-related patterns of anaerobic periodontal bacterial detection in endodontic–periodontal lesions.
- Reduced co-occurrence of selected anaerobic periodontal bacteria in deeper pockets among smokers may indicate that tobacco exposure modifies the expected relationship between pocket depth and microbial distribution.

**Public health implications—What are the key implications or messages for practitioners, policy makers and/or researchers in public health?**

- Smoking status should be considered when interpreting microbiological findings and planning clinical management for patients with endodontic–periodontal lesions.
- Future public health and clinical research should further investigate how tobacco exposure influences oral microbial ecology, periodontal disease progression, and outcomes of integrated endodontic–periodontal therapy.

**Abstract:**

Endodontic–periodontal lesions are complex conditions in which endodontic infection and periodontal breakdown coexist and may create anaerobic microbial niches along the root surface. Although smoking is a well-established modifier of periodontal disease progression and subgingival microbial ecology, its influence on the depth-related distribution of anaerobic periodontal bacteria in teeth affected by endodontic–periodontal lesions remains incompletely understood. This cross-sectional study investigated the distribution patterns and co-occurrence of selected anaerobic periodontal bacteria in smokers and non-smokers with endodontic–periodontal lesions, considering periodontal pocket depth and anatomical site. Subgingival samples were collected from periodontal pockets of different probing depths (3–4 mm, 5–6 mm, and ≥7 mm), as well as from healthy gingival sulci and oral mucosa, in 26 patients with endodontic–periodontal lesions. The presence of *Porphyromonas gingivalis*, *Prevotella intermedia*, *Tannerella forsythia*, *Prevotella nigrescens*, and *Aggregatibacter actinomycetemcomitans* was assessed. Detection was performed using polymerase chain reaction (PCR). Qualitative detection frequencies and microbial co-occurrence patterns were compared between smokers and non-smokers across sites and pocket depths. Non-smokers showed higher detection of *Tannerella forsythia* in pockets ≥ 7 mm (*p* < 0.05). Overall microbial co-occurrence was lower in smokers in deeper periodontal pockets, whereas detection patterns in healthy gingival sulci and oral mucosa were broadly comparable between groups. Our findings suggest that smoking may be associated with an attenuated depth-related detection pattern and reduced co-occurrence of selected anaerobic periodontal bacteria in endodontic–periodontal lesions.

## 1. Introduction

Endodontic–periodontal lesions are complex pathological conditions characterized by the simultaneous involvement of pulpal and periodontal tissues, representing an important diagnostic and therapeutic challenge. These lesions result from anatomical and microbiological communication between the root canal system and periodontal tissues through apical foramina, lateral canals, and dentinal tubules, allowing bidirectional dissemination of microorganisms and inflammatory mediators [1,2]. Their progression may lead to severe attachment loss, alveolar bone resorption, pulpal necrosis, and tooth loss, particularly when diagnosis and treatment are delayed.

The development of these lesions is closely associated with complex subgingival microbial communities, especially Gram-negative anaerobic bacteria, whose interaction with host susceptibility and environmental factors contributes to disease initiation and progression [3]. Their microbiological profile includes species commonly associated with both endodontic infections and periodontal disease, such as *Porphyromonas gingivalis*, *Tannerella forsythia*, *Prevotella intermedia*, *Prevotella nigrescens*, and *Aggregatibacter actinomycetemcomitans*, which are considered relevant microbial markers of disease severity and persistence [4,5].

*Porphyromonas gingivalis* is frequently described as a keystone pathogen because of its capacity to modulate host immune responses and influence the surrounding microbial community. *Tannerella forsythia* and *Prevotella intermedia* have been associated with periodontal tissue destruction and chronic inflammation, while *Prevotella nigrescens* is commonly detected in anaerobic periodontal environments and may coexist with other pathogenic species [6]. *Aggregatibacter actinomycetemcomitans*, although more strongly linked to aggressive forms of periodontitis, may also occur in chronic cases, reflecting the ecological diversity of periodontal microbial communities [7].

Smoking is an independent risk factor for chronic periodontitis and may influence the prognosis of teeth affected by combined endodontic–periodontal infections. Tobacco exposure alters gingival vascularization, host immune responses, tissue oxygenation, and reparative capacity, creating ecological conditions that favor anaerobic bacterial persistence and dysbiosis. Beyond these systemic and immunological effects, smoking may also modify the structure and spatial distribution of subgingival and root-associated microbial communities. Previous studies suggest that smokers tend to exhibit less diverse and more homogeneous microbial profiles, potentially altering the relationship between periodontal pocket depth, pulpal infection, and microbial composition [8].

Periodontal pocket depth is a key clinical parameter in endodontic–periodontal lesions because it reflects periodontal destruction and may indicate communication between periodontal and endodontic compartments. Deeper pockets provide more anaerobic and nutrient-rich environments, generally associated with increased detection of disease-related bacteria [9]. Although non-smokers usually show a depth-dependent increase in anaerobic periodontal pathogens, this gradient may be less evident in smokers, suggesting that tobacco exposure can modify expected microbial distribution patterns [10,11].

Other intraoral sites, such as the gingival sulcus and oral mucosa, may also serve as reservoirs for pathogens involved in endodontic–periodontal lesions. Molecular studies indicate that these sites can harbor bacteria associated with subgingival and endodontic biofilms, contributing to recolonization and disease persistence after periodontal and endodontic therapy. Despite this evidence, few studies have specifically investigated how smoking may influence the microbial profile of endodontic–periodontal lesions. In particular, limited attention has been given to the depth-stratified distribution and co-occurrence patterns of key anaerobic periodontal bacteria across periodontal pockets of different depths, healthy gingival sulci, and oral mucosal sites. This gap is clinically relevant, since smoking-related changes in microbial gradients and interspecies associations within these lesions could challenge conventional pocket depth thresholds used to guide microbiological sampling and treatment decisions in tobacco users.

Therefore, this cross-sectional study investigated the depth-related distribution and co-occurrence of selected anaerobic periodontal bacteria in smokers and non-smokers with endodontic–periodontal lesions, across sites of varying pocket depth, healthy gingival sulci, and oral mucosa.

## 2. Materials and Methods

### 2.1. Patient Selection

This pilot study was approved by the Institutional Research Ethics Committee (protocol number: CAAE 0104.0.189.000-07, approved on 26 March 2013). All patients presented with intact teeth with necrotic pulp tissue, periapical lesions, chronic periodontal disease, and absence of systemic alterations. All samples were collected from periodontal pockets that bled on probing and bone resorption.

The sample size definition of the present study must be interpreted considering the specific clinical nature of endo-periodontal lesions associated with structurally sound teeth, a condition recognized as having low prevalence and difficult clinical recruitment. Unlike conventional chronic periodontitis, these lesions require the simultaneous presence of both periodontal and endodontic involvement in teeth without extensive restorations, root fractures, previous endodontic treatment, or structural alterations that could act as diagnostic confounding factors. This clinical combination substantially reduces patient eligibility and limits the number of cases available for observational studies [1,2].

Participants were classified as smokers or non-smokers based exclusively on self-report. Smokers were defined as individuals who were active smokers at the time of sample collection and reported smoking approximately one pack of cigarettes per day, corresponding to 20 cigarettes, for at least one year. Non-smokers were defined as individuals who reported having never smoked. Former smokers were excluded from the study.

The diagnosis of endodontic–periodontal lesions was established through combined clinical, periodontal, endodontic, and radiographic assessment, according to the 2018 classification proposed by the American Academy of Periodontology and the European Federation of Periodontology. According to the classification criteria for endodontic–periodontal diseases, the lesions included in this study were categorized as primary periodontal lesions with secondary endodontic involvement. Accordingly, periodontal breakdown was considered the primary pathological event, with subsequent retrograde pulpal compromise. All included teeth were structurally sound and had not received previous endodontic treatment, thereby reducing potential diagnostic confounding related to prior therapeutic interventions. Eligible teeth showed concomitant evidence of periodontal and endodontic involvement, including localized periodontal pockets deeper than 3 mm, clinical attachment loss at the affected site, signs of pulpal involvement based on pulp vitality testing and endodontic examination, and radiographic evidence compatible with periapical and/or lateral bone involvement. When present, sinus tracts were clinically traced to support the differential diagnosis, and tooth mobility was recorded [12]. 

Only structurally sound teeth were included, defined as teeth without extensive restorations, previous endodontic treatment, root caries, vertical root fracture, perforations, or other structural alterations that could confound the diagnosis. Root fracture and other non-periodontal/non-endodontic causes of attachment loss were excluded through clinical inspection, periodontal probing pattern, radiographic examination, and complementary diagnostic procedures when necessary. Teeth with isolated periodontal lesions, isolated endodontic lesions, or lesions primarily related to fracture, perforation, or iatrogenic damage were excluded.

Each participant underwent sample collection from three anatomical sites, designated as follows: periodontal pocket; healthy gingival sulcus; oral mucosa. This study was designed as an exploratory cross-sectional investigation aimed at describing microbial distribution patterns rather than testing predefined hypotheses or estimating population parameters.

For determining the prevalence of *Tannerella forsythia*, *Aggregatibacter actinomycetemcomitans*, *Porphyromonas gingivalis*, *Prevotella nigrescens* and *Prevotella intermedia* at different stages of periodontal disease progression, samples from site periodontal pocket were subdivided according to probing depth as follows: periodontal pockets of 3–4 mm; periodontal pockets of 5–6 mm; and periodontal pockets ≥ 7 mm. The bacterial species selected for analysis—*Porphyromonas gingivalis*, *Prevotella intermedia*, *Tannerella forsythia*, *Prevotella nigrescens*, and *Aggregatibacter actinomycetemcomitans*—were chosen based on their consistently high prevalence and recognized pathogenic role in endodontic–periodontal lesions and do not represent the full diversity of the subgingival microbiome. 

All patients presented endodontic–periodontal lesions in sound teeth with periodontal pockets deeper than 3 mm. Exclusion criteria comprised systemic diseases and/or medications that could alter the immune response; use of antibiotics within the last six months; periodontal treatment within the previous 12 months; and the presence of removable dental prostheses and/or orthodontic appliances [13]. Clinical periodontal parameters were used exclusively for site classification and sample stratification according to probing depth. The focus of the study was the qualitative distribution of bacterial detection across sites.

### 2.2. Sample Collection and Storage

Sampling was standardized by the same trained operator after supragingival plaque removal and contamination control. For each patient, samples were collected from multiple anatomical sites, including periodontal pockets (3–4 mm, 5–6 mm, and ≥7 mm), healthy gingival sulci, and oral mucosa. Sterile mouth mirrors, graduated periodontal probes, and absorbent paper points were used. Prior to sampling periodontal pockets and gingival sulci, relative isolation was achieved with cotton rolls, and supragingival biofilm was carefully removed using curettes, cotton pellets, and dental floss to minimize contamination. Subgingival samples were obtained by inserting two sterile absorbent paper points into the selected sites for 20 s and then transferring them to Eppendorf tubes containing 500 µL of sterile saline solution (0.9% NaCl). Oral mucosa samples were collected with sterile swabs and similarly transferred to tubes with sterile saline.

Immediately after collection, all samples were transported to the Microbiology and Immunology Laboratory and stored at freezing temperature until analysis. Subsequently, the material was subjected to molecular detection of *Tannerella forsythia*, *Aggregatibacter actinomycetemcomitans*, *Porphyromonas gingivalis*, *Prevotella nigrescens*, and *Prevotella intermedia* by polymerase chain reaction (PCR) according to their respective anatomical sites. The sampling strategy was designed to capture potential variations in bacterial detection across different anatomical sites and periodontal pocket depths.

### 2.3. Polymerase Chain Reaction (PCR)

In this study, species-specific primer pairs (Table 1) were used for qualitative detection of *Tannerella forsythia*, *Aggregatibacter actinomycetemcomitans*, *Porphyromonas gingivalis*, *Prevotella nigrescens*, and *Prevotella intermedia* by polymerase chain reaction (PCR). The method was selected to assess presence or absence of target species across different anatomical sites and probing depths. We included DNA from standard reference strains as positive controls. DNA extraction was performed using the phenol–chloroform protocol followed by ethanol precipitation. DNA purity was confirmed by spectrophotometric evaluation (Thermo Fisher Scientific, Waltham, MA, USA) and agarose gel visualization (Loccus Biotecnologia, Cotia, SP, Brazil).

PCR amplification reactions were performed in a reaction mixture containing 1·PCR buffer (10 mM Tris-HCl, pH 9.0, 50 mM KCl, and 15 mM MgCl_2_), 200 mM of four deoxynucleotide triphosphates, 12.5 pmol of each primer, 2.5 U Taq DNA polymerase, and 5 mL supernatant from the samples submitted for DNA extraction at a final volume of 50 mL. The reaction mixture was incubated in a thermocycler. Samples were initially denatured at 94_C for 5 min and then submitted to 35 cycles of 60 s at 94_C, 45 s at 67_C, and 30 s at 72_C. A negative control (absence of DNA template), a positive control, and molecular mass marker (100 base pair [bp] DNA ladder) were included in each amplification set. PCR amplification products were analyzed through electrophoresis in a 1.5% agarose gel run in Tris–borate–EDTA buffer, stained with ethidium bromide, and documented under ultraviolet light.

The specification of samples Tf, Aa, Pg, Pi, and Pn was carried out using PCR DNA amplification of a specific sequent of bp for each of the species as described. In complement to the clinical isolates, standard strains were used as a reference: Tf American Type Culture Collection 43037, Aa American Type Culture Collection 29522, Pg American Type Culture Collection 33277, Pi American Type Culture Collection 25611, and Pn National Collection of Type Cultures 9336. Total genomic DNA was obtained using additional RNase treatment. The concentration of DNA was calculated from the measurement (260 nm) in a spectrophotometer at A260, and the quality was estimated by the A260:A280 ratio, electrophoresis in an agarose gel, and comparison with standard DNA.

Quantitative PCR was not employed, as the primary objective of the study was not to assess bacterial load, but rather to describe distribution patterns and co-occurrence of selected periodontal pathogens across sites. 

### 2.4. Statistical Analysis

The primary outcome of the analysis was the difference in microbial co-occurrence in periodontal pockets ≥ 5 mm between smokers and non-smokers. All other analyses were considered secondary and exploratory. The statistical analysis was performed to evaluate both the individual detection of each bacterial species and the overall co-occurrence of microorganisms according to smoking status and anatomical site. Comparisons of bacterial detection frequencies between smokers and non-smokers were performed separately for each anatomical site and, for periodontal pockets, separately within each probing depth category. Fisher’s exact test was used to compare the detection frequency of each bacterial species between smokers and non-smokers within each site/depth category. The Mann–Whitney U test was used to compare the total number of bacterial species detected simultaneously within the same site/depth category. All analyses were two-tailed, and a *p*-value < 0.05 was considered statistically significant. Analyses were performed using Jamovi 2.6.15.

Additionally, the difference in detection prevalence between smokers and non-smokers (Δ% = Y − N) was computed for each bacterial species to construct a color-coded heatmap using a diverging color scale centered at zero, where red denotes a higher prevalence in smokers and blue a higher prevalence in non-smokers. Data visualization was performed in Python version 3.11.5 using the matplotlib version 3.8.0 and seaborn version 0.13.0 libraries. 

## 3. Results

A convenience sample of 26 adult patients of both sexes from 10 (40%) male residents and 16 (60%) female residents of city of Lavras (MG) and the surrounding region, aged 27 to 54 years and clinically diagnosed with endodontic–periodontal lesions through clinical and radiographic examinations. The results are presented emphasizing distribution patterns and microbial co-occurrence across anatomical sites, periodontal pocket depths, and smoking status. The detection frequencies of *Tannerella forsythia*, *Aggregatibacter actinomycetemcomitans*, *Porphyromonas gingivalis*, *Prevotella nigrescens*, and *Prevotella intermedia* in smokers and non-smokers with endodontic–periodontal lesions, stratified by anatomical site and periodontal pocket depth, are presented in Table 2. According to the two-tailed Fisher’s exact test, a statistically significant difference between smokers and non-smokers was observed only for *Tannerella forsythia* in deep periodontal pockets (≥7 mm; *p* = 0.04). Although *Prevotella intermedia* and *Prevotella nigrescens* showed lower detection frequencies in smokers than in non-smokers in 5–6 mm pockets, these differences did not reach statistical significance (*p* = 0.06).

**Figure 1:**
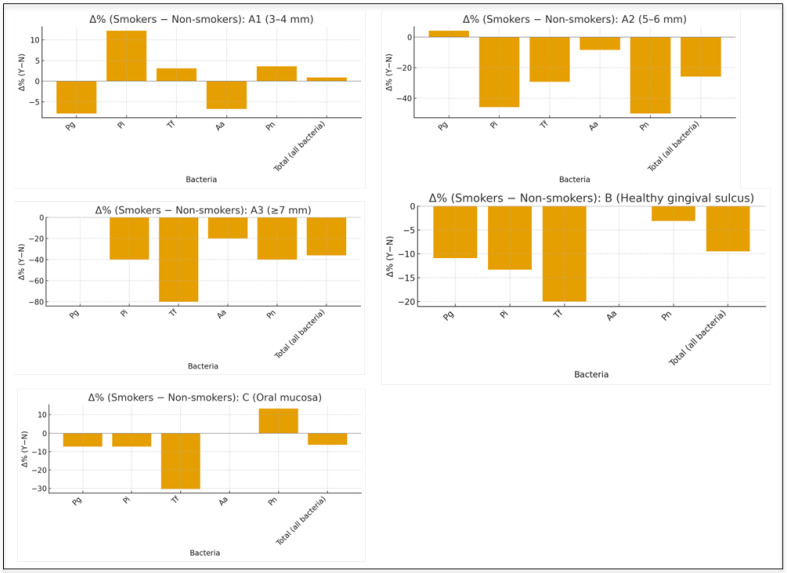
Differences in bacterial detection between smokers and non-smokers across anatomical sites (Δ% = Y − N). A1: periodontal pockets 3–4 mm; A2: 5–6 mm; A3: ≥7 mm; B: healthy gingival sulcus; C: oral mucosa. Bars represent the difference in detection prevalence (%) of each bacterial species between smokers and non-smokers. Positive Δ% values indicate higher prevalence among smokers, whereas negative values indicate higher prevalence among non-smokers. *Porphyromonas gingivalis* (Pg); *Prevotella intermedia* (Pi); *Tannerella forsythia* (Tf); *Aggregatibacter actinomycetemcomitans* (Aa) and *Prevotella nigrescens* (Pn).

The number of microorganisms detected simultaneously per site (co-occurrence) was lower in smokers than in non-smokers in periodontal pockets of 5–6 mm and ≥7 mm, with statistically significant differences observed for both depth categories (*p* = 0.012). These results indicate that smoking is associated with reduced co-occurrence of the selected bacterial species in deeper periodontal pockets, suggesting a less frequent simultaneous detection of target pathogens under smoking exposure (Figure 1). No statistically significant differences in microbial co-occurrence were observed in shallow periodontal pockets (3–4 mm), healthy gingival sulci, or oral mucosa.

**Figure 2:**
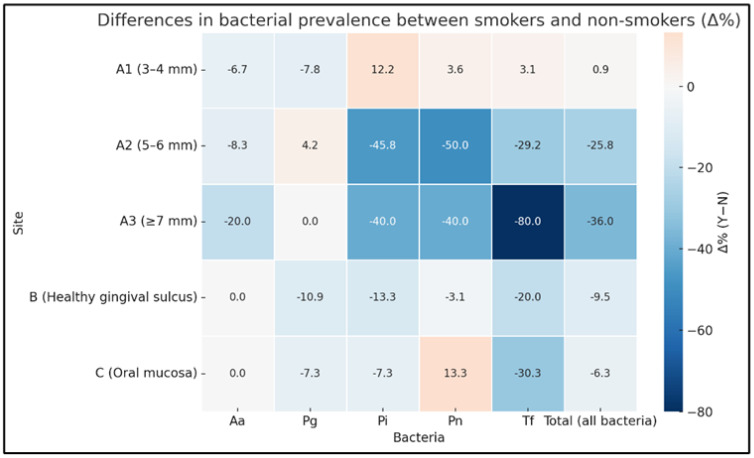
Heatmap showing differences in bacterial detection between smokers and non-smokers across anatomical sites (Δ% = Y − N). Positive values (red shades) indicate a higher prevalence among smokers, while negative values (blue shades) indicate a higher prevalence among non-smokers. The color intensity reflects the magnitude of the difference. A1: periodontal pockets 3–4 mm; A2: 5–6 mm; A3: ≥7 mm; B: healthy gingival sulcus; C: oral mucosa. The heatmap visually summarizes the comparative distribution of *Porphyromonas gingivalis* (Pg), *Prevotella intermedia* (Pi), *Tannerella forsythia* (Tf), *Aggregatibacter actinomycetemcomitans* (Aa), and *Prevotella nigrescens* (Pn) between groups.

The heatmap illustrates distinct qualitative patterns of bacterial detection according to smoking status and periodontal pocket depth (Figure 2). However, in deeper pockets (>5 mm), *Prevotella intermedia* (Pi) and *Prevotella nigrescens* (Pn) were more frequently detected in non-smokers, while *Tannerella forsythia* (Tf) showed reduced prevalence among smokers. 

In healthy gingival sulci and oral mucosa, detection patterns were broadly similar between smokers and non-smokers, with no consistent depth-related trends observed, indicating that the influence of smoking on microbial distribution was more evident in periodontal pockets.

## 4. Discussion

The variability in the detection of the selected anaerobic bacterial species was greater among non-smoking patients compared with smokers, indicating that, in the absence of tobacco exposure, these target pathogens exhibited greater site-related variation. This observation is consistent with sequencing-based studies demonstrating that smokers may exhibit earlier dysbiosis, reduced microbial diversity, and more homogeneous subgingival ecological patterns, although the present study assessed only the qualitative detection of selected bacterial species [8]. This pattern may reflect differences in ecological pressures acting on the oral environment, as exposure to nicotine and other tobacco-derived compounds has been shown to influence bacterial metabolism and modulate host immune responses [9,10]. 

In line with this interpretation, next-generation sequencing studies have demonstrated that smokers harbor a less diverse and more homogeneous subgingival microbiota, characterized by reduced commensal species and predominance of Gram-negative anaerobes, whereas non-smokers show higher diversity and greater inter-site variability [8,14]. Together with the present qualitative PCR findings, these data support the hypothesis that smoking may be associated with a more uniform detection pattern of selected periodontal pathogens, whereas non-smokers may show more evident site- and depth-related variation.

The observation that *Porphyromonas gingivalis* (Pg) was detected at similar frequencies across different probing depths in both smokers and non-smokers suggests that this species exhibits a broad colonization capacity that is relatively independent of pocket depth. This finding is consistent with evidence indicating that Pg demonstrates high diagnostic sensitivity across multiple probing levels, without a strictly linear relationship between depth and prevalence [10]. In this context, Pg may be regarded as a consistently detected target species in the present sample, maintaining its presence across distinct anatomical conditions and smoking status.

Among non-smoking patients, *Tannerella forsythia*, *Prevotella intermedia*, and *Prevotella nigrescens* showed higher detection frequencies in deeper periodontal pockets, suggesting a descriptive depth-related pattern. This pattern is consistent with classical models of periodontitis, in which deeper pockets provide more anaerobic and nutrient-rich conditions that favor the establishment of disease-associated microbial consortia. Supporting this interpretation, Wang et al. [9] reported variations in microbial richness and community structure according to probing depth, with higher relative abundances of anaerobic taxa—particularly *T. forsythia* and *Prevotella* spp.—in sites exceeding 6 mm. In the present study, however, among the species-specific comparisons, only *T. forsythia* in pockets ≥ 7 mm showed a statistically significant difference between smokers and non-smokers. Therefore, the lower detection frequencies of *P. intermedia* and *P. nigrescens* observed in smokers in 5–6 mm pockets should be interpreted as non-significant trends rather than definitive species-specific differences.

In contrast, the depth-related increase in the detection of selected anaerobic species appeared less evident in smokers, suggesting that smoking may attenuate the expected relationship between periodontal pocket depth and microbial detection. In smokers, shallow sites may already harbor disease-associated taxa, thereby reducing the microbiological gradient between shallow and deep pockets. This interpretation is supported by metatranscriptomic evidence demonstrating early functional alterations in the subgingival biofilm of young smokers, even prior to the formation of deep periodontal pockets, indicating accelerated dysbiosis and reduced dependence between microbial profiles and probing depth [11]. Nevertheless, given that most species-specific comparisons did not reach statistical significance, this interpretation should be considered exploratory and hypothesis-generating.

This trend suggests that smoking may be associated with reduced co-occurrence of the selected anaerobic periodontal pathogens, particularly in advanced disease sites. In contrast, healthy gingival sulci and oral mucosa showed broadly similar detection patterns between groups, indicating that the influence of smoking may be more evident in periodontal pockets. Although non-smokers tended to show higher detection frequencies of selected anaerobic species in deeper periodontal pockets, the species-specific results should be interpreted cautiously, since only *T. forsythia* in pockets ≥ 7 mm showed a statistically significant difference between smokers and non-smokers.

The patient selection process in the current study followed the classification of endo-periodontal lesions proposed by the American Academy of Periodontology and the European Federation of Periodontology, which recognizes the diagnostic complexity and lower frequency of these conditions compared with primary periodontal diseases [12]. These characteristics partly explain the relatively small sample size of the present study. Microbiological studies on endo-periodontal lesions frequently include reduced sample sizes, generally ranging from 10 to 30 cases [15,16,17,18,19,20,21]. 

Some methodological considerations should be acknowledged. The relatively small convenience sample reflects the low frequency and diagnostic complexity of endodontic–periodontal lesions in structurally sound teeth, but the strict eligibility criteria contributed to a more homogeneous and clinically well-defined sample. Conventional PCR allowed qualitative detection of selected anaerobic periodontal species rather than bacterial quantification or full microbiome characterization; nevertheless, it was suitable for the exploratory assessment of detection patterns and co-occurrence of clinically relevant target pathogens. Although the cross-sectional design limits causal interpretation, standardized sampling across periodontal pocket depths, healthy gingival sulci, and oral mucosa allowed a focused comparison between smokers and non-smokers. Thus, these findings provide clinically relevant data that smoking may influence the depth-related detection and co-occurrence of selected anaerobic bacteria in endodontic–periodontal lesions. 

From a clinical and scientific perspective, these findings underscore the relevance of considering smoking status when interpreting microbiological patterns in endodontic–periodontal lesions. Periodontal pocket depth remains a meaningful indicator of microbial distribution in non-smokers but may have reduced discriminatory value in smokers. Scientifically, the present results highlight the importance of addressing intraoral variability and site-specific microbial patterns, as well as the need to further investigate how smoking influences the relationship between periodontal pocket depth and bacterial distribution. 

The present findings, obtained through targeted PCR-based detection, should be interpreted in light of NGS-based oral microbiome studies showing that periodontal pocket depth is associated with marked shifts in subgingival microbial composition, with deeper sites favoring more anaerobic and dysbiotic communities. These patterns may extend beyond periodontal pockets, as recent evidence indicates that the tongue dorsum, although microbiologically distinct, may also reflect periodontal status and pocket depth, supporting the concept of the oral cavity as an interconnected microbial ecosystem [22]. The systemic relevance of periodontal pathogens is also noteworthy. Experimental studies have shown that repeated oral exposure to *Porphyromonas gingivalis* may induce bacterial translocation to the hippocampus and trigger neuroinflammatory and neurodegenerative changes consistent with Alzheimer’s disease-like pathology [23]. In the present study, the consistent detection of *P. gingivalis* across pocket depths and smoking groups supports its persistent colonization. Therefore, while the targeted PCR approach used here is useful for detecting selected pathogens, future NGS-based studies including other oral reservoir sites may better characterize the microbial ecosystem associated with endodontic–periodontal lesions in smokers and non-smokers.

Overall, the findings of this study—greater variability in the detection of selected bacterial species in non-smokers, stable detection of *P. gingivalis* across pocket depths, increased detection of *T. forsythia*, *P. intermedia*, and *P. nigrescens* with increasing depth in non-smokers, and a distinct detection pattern in smokers—suggest that smoking may modify the relationship between periodontal pocket depth and the qualitative detection of selected anaerobic periodontal pathogens. 

## 5. Conclusions

In this cross-sectional study, smoking was associated with attenuated depth-related bacterial detection and reduced microbial co-occurrence in periodontal pockets of teeth with endodontic–periodontal lesions, a pattern less evident in non-smokers.

## Figures and Tables

**Table 1:** Primers for PCR identification of *Tannerella forsythia*, *Aggregatibacter actinomycetemcomitans*, *Porphyromonas gingivalis*, *Prevotella intermedia* and *Prevotella nigrescens*.

Species	Primer Sequence (5′-3′)	Base Pairs
*Tannerella forsythia*	59-GGGTGAGTAACGCGTATGTAACCT-39 59-ACCCATCCGCAACCAATAAA-39	127 bp
*Aggregatibacter actinomycetemcomitans*	5′-AAACCCATCTCTGAGTTCTTCTTC-3′ 5′-ATCACCTTGGACTGACATT-3′	449 bp
*Porphyromonas gingivalis*	59-AGGCAGCTTGCCATACTGCG-39 59-ACTGTTAGCAACTACCGATGT-39	443 bp
*Prevotella intermedia*	59-CGGTCTGTTAAGCGTGTTGTG-39 59-CACCATGAATTCCGCATACG-39	99 bp
*Prevotella nigrescens*	59-CAGCCAAACACGATACCTGTTG-39 59-TTCCATTGGACACATCAGCATT-39	150 bp

**Table 2:** Detection frequency of anaerobic bacteria according to smoking status.

Site	Bacteria	Non-Smokers (N)	Smokers (Y)	Δ% (Y − N)	*p*-Value
Periodontal Pocket 3–4 mm	Pg	53.3% (8/15)	45.5% (5/11)	−7.8	1.00
	Pi	33.3% (5/15)	45.5% (5/11)	12.2	0.69
	Tf	33.3% (5/15)	36.4% (4/11)	3.1	1.00
	Aa	6.7% (1/15)	0.0% (0/11)	−6.7	1.00
	Pn	60.0% (9/15)	63.6% (7/11)	3.6	1.00
	Overall detection	37.3% (28/75)	38.2% (21/55)	0.9	1.00
Periodontal Pocket 5–6 mm	Pg	58.3% (7/12)	62.5% (5/8)	4.2	1.00
	Pi	83.3% (10/12)	37.5% (3/8)	−45.8	0.06
	Tf	66.7% (8/12)	37.5% (3/8)	−29.2	0.36
	Aa	8.3% (1/12)	0.0% (0/8)	−8.3	1.00
	Pn	75.0% (9/12)	25.0% (2/8)	−50.0	0.06
	Overall detection	58.3% (35/60)	32.5% (13/40)	−25.8	0.01 ^#^
Periodontal Pocket ≥7 mm	Pg	60.0% (3/5)	60.0% (3/5)	0.0	1.00
	Pi	80.0% (4/5)	40.0% (2/5)	−40.0	0.52
	Tf	100.0% (5/5)	20.0% (1/5)	−80.0	0.04 *
	Aa	20.0% (1/5)	0.0% (0/5)	−20.0	1.00
	Pn	80.0% (4/5)	40.0% (2/5)	−40.0	0.52
	Overall detection	68.0% (17/25)	32.0% (8/25)	−36.0	0.02 ^#^
Healthy gingival sulcus	Pg	20.0% (3/15)	9.1% (1/11)	−10.9	0.61
	Pi	13.3% (2/15)	0.0% (0/11)	−13.3	0.49
	Tf	20.0% (3/15)	0.0% (0/11)	−20.0	0.24
	Aa	0.0% (0/15)	0.0% (0/11)	0.0	1.00
	Pn	66.7% (10/15)	63.6% (7/11)	−3.1	1.00
	Overall detection	24.0% (18/75)	14.5% (8/55)	−9.5	0.27
Oral mucosa	Pg	80.0% (12/15)	72.7% (8/11)	−7.3	1.00
	Pi	80.0% (12/15)	72.7% (8/11)	−7.3	1.00
	Tf	66.7% (10/15)	36.4% (4/11)	−30.3	0.23
	Aa	0.0% (0/15)	0.0% (0/11)	0.0	1.00
	Pn	86.7% (13/15)	100.0% (11/11)	13.3	0.49
	Overall detection	62.7% (47/75)	56.4% (31/55)	−6.3	0.48

Δ% (Y−N) represents the difference in prevalence between smokers and non-smokers. * Statistically significant difference between smokers and non-smokers according to Fisher’s exact test, considering the detection of each bacterial species at each anatomical site. # Statistically significant difference according to the Mann–Whitney test, considering the total co-occurrence of microorganisms at each site. Abbr.: *Porphyromonas gingivalis* (Pg); *Prevotella intermedia* (Pi); *Tannerella forsythia* (Tf); *Aggregatibacter actinomycetemcomitans* (Aa) and *Prevotella nigrescens* (Pn).

## Data Availability

The data supporting the findings of this study are available from the corresponding author upon reasonable request. The data are not publicly available due to privacy and ethical restrictions related to clinical information from human participants.

## Abbreviations

The following abbreviations are used in this manuscript:

Aa	*Aggregatibacter actinomycetemcomitans*
APC	Article processing charge
bp	Base pairs
DNA	Deoxyribonucleic acid
dNTP	Deoxynucleotide triphosphate
EDTA	Ethylenediaminetetraacetic acid
PCR	Polymerase chain reaction
Pg	*Porphyromonas gingivalis*
Pi	*Prevotella intermedia*
Pn	*Prevotella nigrescens*
Tf	*Tannerella forsythia*
TBE	Tris–borate–EDTA
Δ%	Difference in detection prevalence between smokers and non-smokers
